# Local Effects of Regulatory T Cells in MUC1 Transgenic Mice Potentiate Growth of MUC1 Expressing Tumor Cells *In Vivo*

**DOI:** 10.1371/journal.pone.0044770

**Published:** 2012-09-17

**Authors:** Daisuke Sugiura, Kaori Denda-Nagai, Mitsuyo Takashima, Ryuichi Murakami, Shigenori Nagai, Kazuyoshi Takeda, Tatsuro Irimura

**Affiliations:** 1 Laboratory of Cancer Biology and Molecular Immunology, Graduate School of Pharmaceutical Sciences, the University of Tokyo, Tokyo, Japan; 2 Department of Microbiology and Immunology, Keio University School of Medicine, Tokyo, Japan; 3 Department of Immunology, Juntendo University School of Medicine, Tokyo, Japan; University of Pittsburgh, United States of America

## Abstract

MUC1 transgenic (MUC1.Tg) mice have widely been used as model recipients of cancer immunotherapy with MUC1. Although MUC1.Tg mice have previously been shown to be immunologically tolerant to MUC1, the involvement of regulatory T (Treg) cells in this phenotype remains unclear. Here, we showed that numbers of Treg cells in MUC1-expressing tumors were greater in MUC1.Tg mice than in control C57BL/6 (B6) mice, and that the growth of tumor cells expressing MUC1, but not that of control cells, in MUC1. Tg mice was faster than in B6 mice. The MUC1.Tg mice appeared to develop MUC1-specific peripheral tolerance, as transferred MUC1-specific T cells were unable to function in MUC1.Tg mice but were functional in control B6 mice. The suppressive function of CD4^+^CD25^high^ cells from MUC1.Tg mice was more potent than that of cells from control B6 mice when Treg cell activity against MUC1-specific T cells was compared *in vitro*. Therefore, the enhanced growth of MUC1-expressing tumor cells in MUC1.Tg mice is likely due to the presence of MUC1-specific Treg cells.

## Introduction

The immune system suppresses or enhances the growth of malignant cells in a variety of ways. However, the role of tumor antigen-specific T cells at the site of tumor growth is not thoroughly understood. In contrast to the known anti-tumor effector functions mediated by tumor antigen-specific T cells, regulatory T (Treg) cells, consisting of a subpopulation of CD4^+^ T cells, are known to be involved in the establishment and growth of tumor cells [1]. Treg cells express high levels of the T cell activation marker, CD25, and Foxp3, a master regulator gene required for the development and function of Treg cells, and suppress the activation and effector function of a wide variety of immune cells by secreting the inhibitory cytokines, IL-10 and TGF-β, and expressing inhibitory proteins, such as CTLA-4 [2]. It has been reported that Treg cells accumulate in tumor tissues, and the number of Treg cells negatively correlates with the survival of cancer patients [3]. There are also many reports showing that the depletion of Treg cells or neutralization of Treg cell-associated molecules suppresses tumor growth *in vivo* [4]. These results clearly demonstrate that Treg cells suppress anti-tumor immune responses and enhance tumor growth. Whether Treg cells recognize a specific tumor antigen and suppress tumor antigen-specific immune responses is an important issue to further understand the involvement of Treg cells in tumor immunity. Importance of specific antigens in the development and activation of Treg cells have previously been shown [5], [6], [7]. However, model antigens and T cells from model antigen-specific TCR transgenic mice are used to simplify the system. There are only few reports focusing on clinically relevant and well-characterized tumor-antigens or on animal models with natural variations of TCRs [8], [9].

In the present study, we used MUC1 as a tumor antigen. Carcinoma-associated MUC1 is known to be recognized by the immune system in cancer patients [10], [11], [12], and MUC1-specific cytotoxic T lymphocytes (CTLs) have been detected in colon, pancreatic, and breast carcinoma patients [13], [14]. Survival of breast and pancreatic carcinoma patients who had antibodies specific for MUC1 was longer than the survival of those without MUC1-specific antibodies [15], [16]. Therefore, MUC1 is widely accepted as an effective target for cancer immunotherapy. MUC1 transgenic (MUC1.Tg) mice, which were generated to investigate the optimal use of MUC1 in cancer immunotherapy [17], are known to be immunologically tolerant to MUC1. The human MUC1 promoter drives the expression of MUC1 in MUC1.Tg mice, and the spatial and temporal patterns of MUC1 expression are very similar to those in humans. Deletion of MUC1-specific T cells in the thymus, called central tolerance, is predicted as one of the mechanism for the ineffective MUC1-specific T cell responses in MUC1.Tg mice, based on the knowledge from Her-2 and CEA transgenic mouse models [18], [19]. However, it was reported that MUC1-specific tolerance was mediated not only by the central but also other peripheral mechanisms [20], and that the presence of MUC1-specific Treg cells has previously been shown in MUC1.Tg mice vaccinated with MUC1 peptide [21].

We found that growth of tumor cells expressing MUC1 was enhanced in MUC1.Tg mice, and the effect was likely due to the local effects of Treg cells. Such enhancement was a result of increased numbers of Treg cells in tumor tissues expressing MUC1. Furthermore, we were able to show, for the first time, that MUC1-specific peripheral tolerance operates in MUC1.Tg mice to support the tumor growth *in vivo*. We also showed that the suppression of MUC1-specific effector function appeared to be mediated by Treg cells. These findings should provide a foundation for the development of effective cancer immunotherapy.

## Results

### Tumor Cells Expressing MUC1 Grow Faster in MUC1.Tg Mice than in B6 Mice

**Figure 1:**
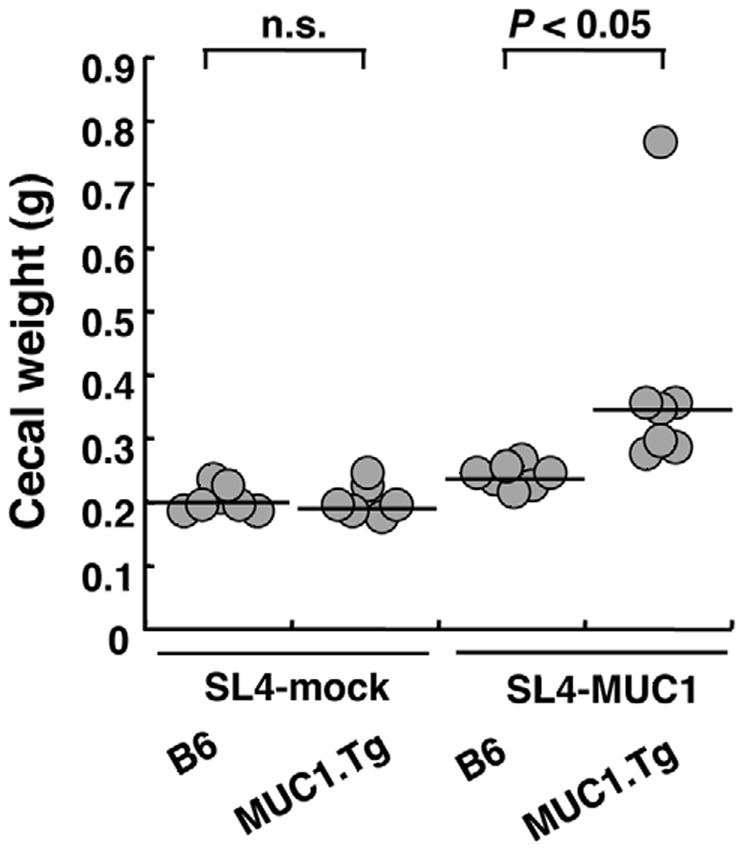
SL4-MUC1 cells grew faster in MUC1.Tg mice than in B6 mice. SL4-mock cells or SL4-MUC1 cells (1×10^6^ cells/50 µL) were injected into the space under the cecal serosa of B6 mice or MUC1.Tg mice. Two weeks after injection, mice were sacrificed, and the weight of the cecum was measured. Lines indicate the median values. Statistical analysis was performed using the Mann-Whitney U-test (n.s.: not significant). A representative result from three independent experiments is shown.

MUC1.Tg mice have previously been shown to be immunologically tolerant to MUC1 when the immune response to B16 melanoma cells transfected with MUC1 cDNA was investigated [17], [22]. In humans, colon carcinoma cells, but not normal colonic epithelia, express MUC1, and the level of MUC1 in colon carcinoma tissues correlates with the stage of the disease [22]. Thus, we tested whether expression of MUC1 influences the growth of colon carcinoma cells in MUC1.Tg mice in the orthotopic transplantation model. We generated *MUC1*-transfected mouse colon carcinoma SL4 cells (SL4-MUC1 cells), which are highly tumorigenic and metastatic in C57BL/6 (B6) mice, and control cells transfected with the empty vector (SL4-mock cells) that exhibited similar growth behavior to the SL4-MUC1 cells. B6 or MUC1.Tg mice were orthotopically injected with SL4-mock or SL4-MUC1 cells into the ceca and sacrificed 2 weeks after the injection. Tumor growth was evaluated by determining the weight of each cecum. When SL4-mock cells were injected, there was no significant difference between B6 and MUC1.Tg mice in the weight of cecum (Fig. 1). However, the weight of cecum induced by SL4-MUC1 cells was heavier when the cells were injected into MUC1.Tg mice than when they were injected into B6 mice (Fig. 1), suggesting that the growth of MUC1-positive tumor cells was enhanced in MUC1.Tg mice. Alternatively, growth of MUC1-positive tumor cells may be suppressed in B6 mice, whereas this suppression was absent in MUC1.Tg mice. To assess such possibility, we analyzed the type of T cells infiltrating in tumor tissues in MUC1.Tg mice.

### The Number of Treg Cells Increases when MUC1-expressing Tumor Cells are Transplanted into MUC1.Tg Mice

**Figure 2:**
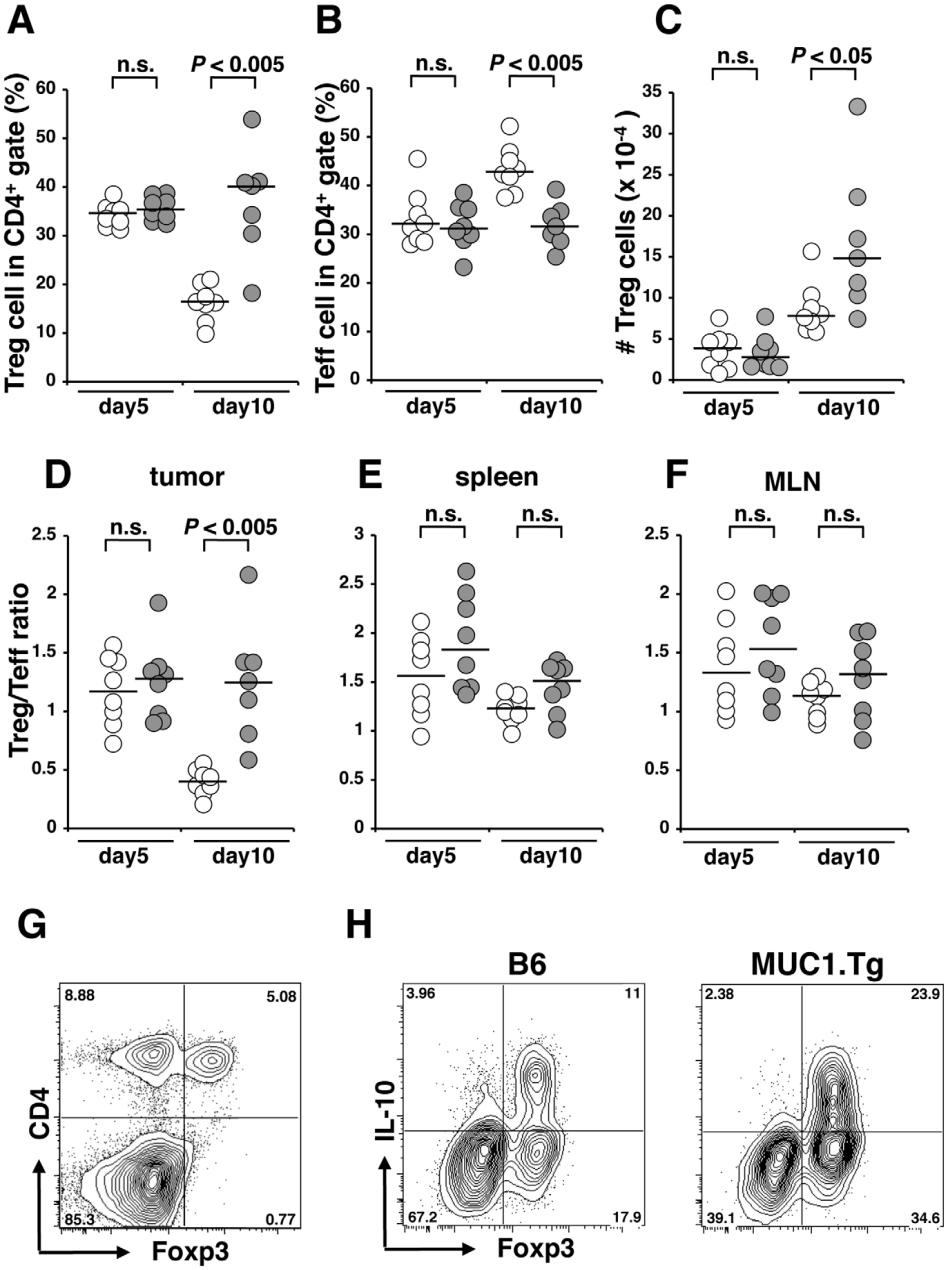
The numbers of Treg cells increased in the tumor tissues in MUC1.Tg mice. SL4-MUC1 cells (1×10^6^ cells/50 µL) were injected into the space under the cecal serosa of B6 mice or MUC1.Tg mice. Five or ten days after injection, mice were sacrificed, the cecal tumors, spleens, and MLNs were resected and single cell suspensions were prepared. (A–G) Cells were stained with CD4, CD25, and Foxp3 and analyzed by flow cytometry. CD4^+^CD25^+^Foxp3^+^ cells were considered Treg cells and CD4^+^CD25^+^Foxp3^−^ cells were considered Teff cells. (A, B) Percentages of Treg cells (A) and Teff cells (B) in CD4^+^ cells in the tumor tissues are shown. (C) Absolute numbers of Treg cells in the tumor tissues are shown. (D–F) Treg/Teff ratios in tumor tissues (D), spleens (E) and MLNs (F) are calculated and shown. Data from B6 mice are indicated as open circles, and data from MUC1.Tg mice are indicated as filled circles. Lines indicate the median values. (G) A representative profile of CD4 and Foxp3 of cells from cecal tumor in MUC1.Tg mice is shown. (H) Cells from tumor tissues in B6 or MUC1.Tg mice were stimulated by PMA and ionomycin for 4 hours and stained with CD4, Foxp3, and IL-10. The cells in CD4^+^ gate were shown. Statistical analysis was performed using the Mann-Whitney U-test (n.s.: not significant). A representative result from three independent experiments (A–G) or two independent experiments (H) is shown.

Because the immunological status of MUC1.Tg mice injected with tumor cells expressing MUC1 has not been thoroughly characterized, we compared T cell subpopulations in B6 and MUC1.Tg mice, particularly the CD4^+^ effector T cells and Treg cells that infiltrated the tumor. SL4-MUC1 cells were orthotopically injected into the ceca of B6 or MUC1.Tg mice, and mice were sacrificed 5 or 10 days later. Cells obtained from cecal tumor tissues were stained for CD4, CD25, and Foxp3 and analyzed by flow cytometry. Percentages of CD4^+^CD25^+^Foxp3^+^ cells (Treg cells) and CD4^+^CD25^+^Foxp3^−^ effector T cells (Teff cells) in the CD4^+^ gate were calculated and are shown in Fig. 2A and B. The percentages of tumor infiltrating Treg and Teff cells 5 days after injection of tumor cells were similar between B6 and MUC1.Tg mice, whereas percentages of tumor infiltrating Treg cells in MUC1.Tg mice were higher than those in B6 mice when the tumor tissues was examined 10 days after injection of tumor cells (Fig. 2A). Moreover, the percentages of tumor infiltrating Teff cells in MUC1.Tg mice were smaller than those in B6 mice at this time point (Fig. 2B). Because the number of intratumoral Treg cells increased at day 10, and the increase was more prominent in MUC1.Tg mice than in B6 mice (Fig. 2C), the enhanced percentages of Treg cells in MUC1.Tg mice at day 10 (Fig. 2A) could be due at least in part to the infiltration or proliferation of Treg cells in the MUC1-expressing tumor tissues. To clarify whether the increase in the number of Treg cells was unique to the tumor tissues, splenocytes and cells from mesenteric lymph nodes (MLNs) were collected from the tumor-bearing mice, and CD4^+^ cells from these organs were analyzed by flow cytometry. The Treg/Teff ratios were calculated and are shown in Fig. 2D–F. Treg/Teff ratios at the tumor site in MUC1.Tg mice were significantly greater than those in B6 mice 10 days after injection of tumor cells. Difference in the ratio between MUC1.Tg and B6 mice was not observed in the spleen or MLNs at day 5 or day 10. These results indicate that increases in the number of Treg cells in MUC1.Tg mice occurred in tissues where cells expressing MUC1 were localized. In our experimental system, almost all of the Foxp3^+^ cells were CD4^+^ (Fig. 2G). To assess whether these cells function as Treg cells, cells obtained from tumor tissues were stimulated by PMA and ionomycin and their capacity to produce IL-10 was evaluated by flow cytometry. Tumors grown in MUC1.Tg mice contained a greater number of CD4^+^Foxp3^+^IL-10^+^ Treg cells than in B6 mice (Fig. 2H). Suppression of anti-tumor immune response by IL-10 may allow the rapid tumor growth observed in MUC1.Tg mice.

### Treg Cells in MUC1.Tg Mice Proliferated in Tumor Tissues

**Figure 3:**
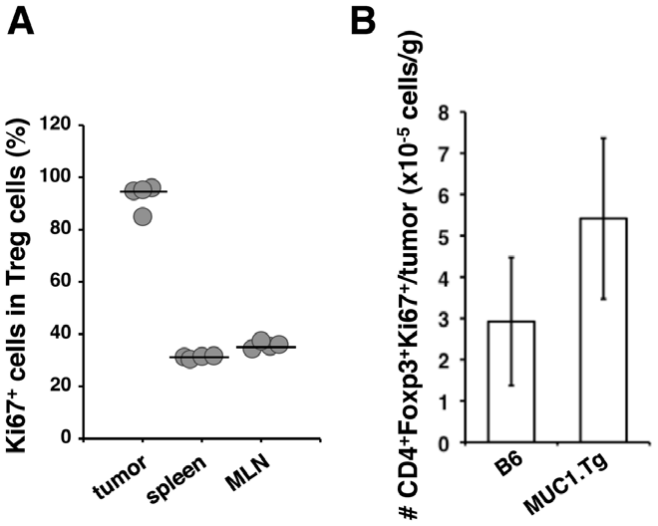
Treg cells were found to proliferate in tumors in MUC1.Tg mice. SL4-MUC1 cells (1×10^6^ cells/50 µL) were injected into the space under the cecal serosa of B6 mice or MUC1.Tg mice. Ten days after injection, mice were sacrificed, the cecal tumors, spleens, and MLNs were resected and single cell suspensions were prepared. (A) Cells from MUC1.Tg mice in each organ were stained with CD4, Foxp3, and Ki-67 and percentage of Ki-67^+^ Treg cells in each organ were shown. Lines indicate the median values. (B) Cells from tumor tissues in B6 or MUC1.Tg mice were stained with CD4, Foxp3, and Ki-67 and the absolute number of Ki-67^+^ Treg cells was calculated. The absolute number of Ki-67^+^ Treg cells normalized to the cecal weight was illustrated. A representative result from two independent experiments is shown.

To further characterize the tumor infiltrating Treg cells, they were tested for Ki-67 antigen, a nuclear protein known to be associated with proliferating cells. Almost all of the Treg cells in tumor tissues were positive with Ki-67. In contrast, Treg cells obtained from MLNs and spleens showed lower percentage of Ki-67^+^ cells than those from tumor tissues (Fig. 3A). Furthermore, the number of tumor infiltrating Ki-67^+^ Treg cells normalized to tumor weights in MUC1.Tg mice was apparently greater than that in B6 mice (Fig. 3B), presumably due to the presence of MUC1 in the tumor microenvironment. Combined with the data in Fig. 2, these results strongly suggest that increased number of Treg cells in the tumor tissues in MUC1.Tg mice is due to the local proliferation of these cells but not to the migration of Treg cells from other lymphoid organs.

### MUC1.Tg Mice Develop MUC1-specific Peripheral Tolerance as Demonstrated by the Suppression of MUC1-specific T Cell Functions

**Figure 4:**
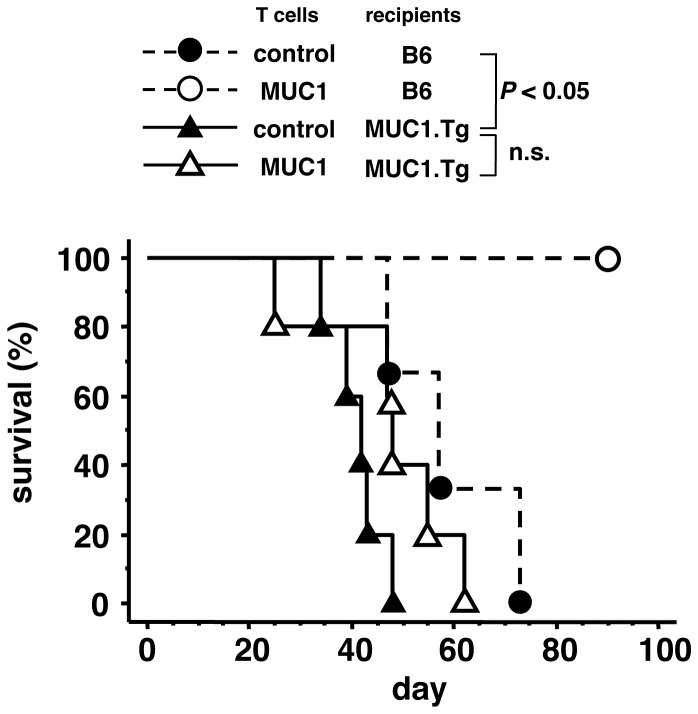
MUC1.Tg mice appeared to elicit MUC1-specific peripheral tolerance. T cells from mice vaccinated with MUC1 DNA or T cells from mice immunized with control vectors (3×10^6^ cells) were mixed with B16-F10-MUC1 cells (5×10^4^ cells) and subcutaneously injected into naïve B6 or MUC1.Tg mice. Survival rates of the mice are shown. Statistical analysis was performed using the log rank test (n.s.: not significant). A representative result from three independent experiments is shown.

The experiments above suggest that Treg cells are involved in tolerance to MUC1 in MUC1.Tg mice. However, we cannot eliminate the possibility that MUC1-specific tolerance is established primarily by the deletion of MUC1-specific T cells in the thymus as shown in experimental systems using other transgenic mice [18], [19]. To examine the importance of Treg cell-mediated peripheral tolerance in MUC1.Tg mice, we performed the Winn assay. MUC1-specific T cells were induced by intradermal immunization of plasmid DNA containing full-length *MUC1* cDNA in B6 mice and isolated from the spleens as described in the Materials and Methods. This protocol was previously shown to activate CD4^+^ MUC1-specific T cells but not CD8^+^ T cells [23], [24]. The MUC1-specific T cells were mixed with B16-F10 melanoma cells transfected with *MUC1* cDNA (B16-F10-MUC1) and subcutaneously injected into naïve B6 or MUC1.Tg mice. The tumor incidences and survival rates in these mice were investigated. B6 mice inoculated with B16-F10-MUC1 cells and MUC1-specific T cells completely rejected the melanoma cells, and all of the mice survived (Fig. 4). In contrast, all of the MUC1.Tg mice died even though they received numbers of MUC1-specific T cells that resulted in 100% survival in B6 mice. The survival curves were very similar to those of mice injected with control T cells. These results clearly indicate that MUC1.Tg mice develop MUC1-spcific peripheral tolerance possibly mediated by Treg cells, and this tolerance mechanism is involved in the escape of tumor cells from elimination by specific T cells.

### Treg Cells from MUC1.Tg Mice Elicit Suppression of MUC1-specific CD4^+^ T Cells *in vitro*

**Figure 5:**
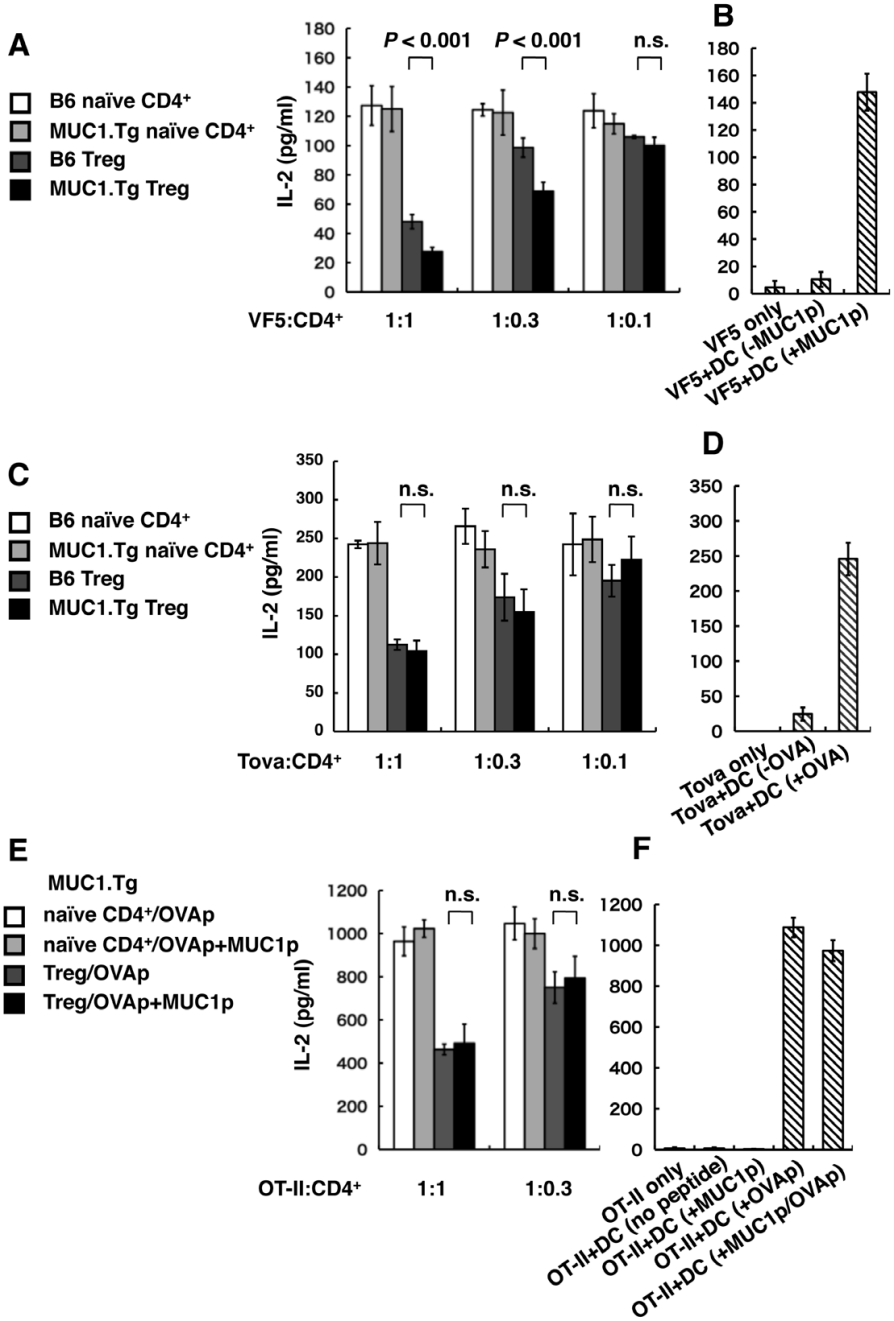
Treg cells in MUC1.Tg mice contained MUC1-specific populations. Antigen specificity of Treg cell suppression was investigated *in vitro*. (A) MUC1-specific T cell hybridoma VF5 cells (1×10^5^ cells) were co-cultured with BM-DCs (2×10^4^ cells) pulsed with MUC1 peptide and CD4^+^ T cells as indicated. (B) VF5 cells (1×10^5^ cells) were cultured alone or co-cultured with BM-DCs (2×10^4^ cells) pulsed with or without MUC1 peptide (+/−MUC1p). (C) OVA-primed CD4^+^ T cells (1×10^5^ cells) were co-cultured with BM-DCs (2×10^4^ cells) pulsed with OVA protein and CD4^+^ T cells as indicated. (D) OVA-primed CD4^+^ T cells (1×10^5^ cells) were cultured alone or co-cultured with BM-DCs (2×10^4^ cells) pulsed with or without OVA (+/−OVA). (E) OVA-specific OT-II T cells (4.5×10^4^ cells) were co-cultured with BM-DCs (4.5×10^3^ cells) pulsed with OVA peptide and/or MUC1 peptide and CD4^+^ T cells obtained from MUC1.Tg mice as indicated. (F) OT-II T cells (4.5×10^4^ cells) were cultured alone or co-cultured with BM-DCs (4.5×10^3^ cells) pulsed with OVA peptide and/or MUC1 peptide. Twenty-four hours (A–D) or 48 hours (E, F) after the initiation of co-cultures, supernatants were collected, and the concentration of IL-2 was measured by ELISA. Data are shown as the mean±SD (n = 3 or 4). Statistical analysis was performed using the Student’s t-test (n.s.: not significant). A representative result from three independent experiments is shown.

The data shown in Fig. 2–4 collectively demonstrated that MUC1.Tg mice had peripheral tolerance to MUC1 and that Treg cells seemed to be involved. Thus, we attempted to confirm that Treg cells from MUC1.Tg mice suppressed MUC1-specific T cell responses *in vitro*. VF5 cells, 13-mer MUC1 peptide-specific CD4^+^ T cell hybridoma cells, have been shown to produce IL-2 when they are co-cultured with bone marrow derived dendritic cells (BM-DCs) pulsed with MUC1 peptides [25]. Using these cells, antigen-specific responses of Treg cells from MUC1.Tg mice were evaluated. CD4^+^CD25^high^ Treg cells were obtained from the spleens and lymph nodes of naïve MUC1.Tg or B6 mice by cell sorting as described in the Materials and Methods. CD4^+^CD25^−^ naïve T cells were used as control cells. Treg or control CD4^+^CD25^−^ T cells were added to a co-culture of VF5 cells and BM-DCs at different ratios, and the concentration of IL-2 in the supernatant was measured by ELISA (Fig. 5A and B). As expected, control CD4^+^CD25^−^ T cells from B6 and MUC1.Tg mice did not show any effect on IL-2 production by VF5 cells. Treg cells from B6 mice suppressed IL-2 production in a cell number-dependent manner. Treg cells obtained from MUC1.Tg mice showed significantly greater suppressive capacity than Treg cells from B6 mice. When we tested Treg cell function against OVA-primed CD4^+^ T cells, Treg cells from both MUC1.Tg and B6 mice showed similar suppressive capacity (Fig. 5C and D). Therefore, MUC1-specific populations seem to be present in CD4^+^CD25^high^ Treg cells obtained from MUC1.Tg mice. To further analyze suppressive mechanism, Treg cells from MUC1.Tg mice were co-cultured with OT-II T cells and BM-DCs pulsed with MUC1 peptide and/or OVA peptide. Treg cells from MUC1.Tg mice suppressed IL-2 production from OT-II T cells co-cultured with OVA pulsed BM-DCs in cell number dependent manner, and enhanced suppression was not observed even when BM-DCs were pulsed with a mixture of OVA and MUC1 peptides (Fig. 5E and F). Thus, the MUC1-specific element of the suppression of IL-2 production by Treg cells from MUC1.Tg mice does not seem to be mediated by bystander effects.

## Discussion

Treg cells are known to play a vital role in the control of growth and rejection of tumor cells. However, the importance of tumor antigen and antigen specificity in Treg cell-mediated suppression in non-TCR transgenic animal remained unclear. The present study reveals that MUC1-specific peripheral tolerance, which supports the growth of tumor cells expressing MUC1, plays an important role in MUC1.Tg mice. The mechanism of this MUC1-specific peripheral tolerance appeared to be due to the presence of MUC1-specific Treg cells. We observed increases in the number of Treg cells in the tumor tissues, but not in the spleens or MLNs, where the presentation of tumor-antigen by antigen-presenting cells should take place due to the close location to tumor site. Furthermore, the higher percentage of Treg cells proliferated in tumor tissues than lymphoid tissues. These results suggest that tumor tissue-specific increases in Treg cells are not due to the migration and accumulation of Treg cells from other lymphoid tissues but also to specific proliferation of Treg cells in tumor tissues possibly responding to MUC1. Alternatively, naïve T cells might be converted into Foxp3^+^ Treg cells in the tumor microenvironment. Such proliferating Treg cells produced IL-10 as shown in Fig. 2H. As a consequence, tumor cell proliferation could be supported by increasing number of Treg cells, which potentially underwent expansion in MUC1.Tg mice in accordance with proliferation of tumor cells expressing MUC1.

The data shown in Fig. 1, 2, 3 collectively suggested that Treg cells played a pivotal role in MUC1-specific tolerance in MUC1.Tg mice. To confirm the importance of MUC1-specific peripheral tolerance in another animal model, we transferred MUC1-specific CD4^+^ T cells into MUC1.Tg mice as shown in Fig. 4. In this experimental system, we achieved to exclude the possible involvement of central tolerance, which was suggested to be the major mechanism to establish the tumor antigen-specific tolerance [18], [19], because functional MUC1-specific T cells were transferred from B6 mice [24]. Tolerance to MUC1, suppression of MUC1-specific T cells, and promotion of tumor growth were observed when MUC1.Tg mice were used as recipients. We believe that the present report is the first to show that peripheral tolerance in MUC1.Tg mice supports the tumor growth *in vivo*. Significance of the involvement of Treg cells in MUC1-specific peripheral tolerance remains to be further confirmed by other types of *in vivo* approaches.

The data from Fig.1, 2, 3, 4 indicated that MUC1-specific peripheral tolerance was maintained by Treg cells. There were some reports addressing the involvement of Tregs in MUC1-specific tolerance in MUC1 Tg mice [20], [21], [26], however antigen specific element in the Treg function was not previously explored well. Our attempt to examine the MUC1 specificity of Treg cells led us to an interesting observation. Treg cells obtained from naïve MUC1.Tg mice, which have a wide variety of TCRs, more strongly suppressed MUC1-specific immune responses *in vitro* than those obtained from B6 mice did. The presence of MUC1-specific Treg cells was previously shown in MUC1.Tg mice vaccinated with MUC1 peptide [21]. Therefore, taking our findings into consideration, it is possible that immunization with MUC1 peptides and transplantation of MUC1-expressing tumor cells activate and induce the proliferation of MUC1-specific Treg cells. Because we used MUC1.Tg mice, which had intact TCRs as discussed above, it remained to be determined whether very few numbers of antigen-specific Treg cells, as observed in our present study, were enough to suppress antigen-specific immune responses *in vivo*. In accord with other reports, our experiments showed that Treg cells elicited suppression in *in vitro* assay systems not only in an antigen dependent but also antigen independent manner [27]. It has been suggested that so many mechanisms are involved in Treg cell mediated suppression [2], though most of these studies were performed based on the notion that Treg cells were antigen independent. In our *in vitro* assays, MUC1-specific Treg cells suppressed IL-2 production by MUC1-specific T cells but not by OVA-specific T cells even though antigen-presenting cells presented both MUC1 and OVA, suggesting that the suppression was mediated not through bystander effects but rather through competition for MUC1 peptide presented by antigen-presenting cells. As shown in Fig. 2H, the number of Treg cells, which produce IL-10, increases in tumor tissues. The microenvironment rich in IL-10 was likely to promote tumor growth. However, the role of MUC1-specific Treg cells in antigen-dependent suppression remains to be determined by *in vivo* experiments.

It was widely accepted that not only CTLs but also tumor antigen-specific CD4^+^ T cells participated in the anti-tumor immune responses through a variety of mechanisms [25]. We also showed that MUC1-specific CD4^+^ T cells played critical roles in the rejection of MUC1-expressing colon carcinoma cells in B6 mice vaccinated with MUC1 cDNA [23], [24]. Antigen-specific CD4^+^ T cells were known to help the induction and maintenance of effector/memory CD8^+^ CTLs [28], [29] and also elicit direct cytotoxic activity against target tumor cells [30], [31]. Therefore, we believe that our findings that MUC1-specific Treg cells suppress IL-2 production from MUC1-specific CD4^+^ T cells provide important information in tumor immunity.

In the present report, antigen-specific Treg cells were shown to support tumor growth by suppressing antigen-specific T cells. Many reports have previously indicated that Treg cells enhance tumor growth [1], [4], yet the mechanism was still not completely understood. Here, we showed for the first time, that MUC1-specific peripheral tolerance operates in MUC1.Tg mice and supports the tumor growth *in vivo*. We also showed that the suppression of MUC1-specific immune response appears to be mediated by Treg cells. It is also important to note that we performed experiments focusing on tumor antigen MUC1 and MUC1.Tg mice with intact wide variety of TCR repertoires. The possibility that tumor-associated MUC1 is aberrantly glycosylated [12] has widely been discussed, and T cells can distinguish glycosylated from non-glycosylated peptides presented on MHC molecules [20], [32]. Whether Treg cells distinguish differentially glycosylated MUC1 will be another important topic for future studies.

## Materials and Methods

### Mice

Specific pathogen-free B6 mice were obtained from CLEA Japan, Inc. MUC1.Tg mice on a B6 background [17] were kindly provided by Dr. Sandra J. Gendler of Mayo Clinic Arizona. Mice were housed under specific pathogen-free conditions and handled according to the guidelines of the Bioscience Committee of the University of Tokyo.

### Construction of *MUC1* cDNA

The full-length human *MUC1* cDNA containing 22 tandem repeats was originally provided by Dr. Olivera J. Finn in the pDKOF vector [33]. The cDNA was excised and recloned into the *Hind*III site of the pCEP4 vector, which includes the CMV promoter (pCEP4-MUC1). The plasmid without the *MUC1* cDNA (pCEP4) was used as a control. The pCEP4 and pCEP4-MUC1 plasmids were amplified in the *E. coli* strain, JM109, and purified using Qiagen Mega-Plasmid columns (Qiagen, Inc.).

### Tumor Cells

SL4, a highly metastatic variant to the liver of the colon 38 murine colon carcinoma cell line [34], was transfected with human *MUC1* cDNA (pDKOF-MUC1) by lipofection using DOTAP Liposomal Transfection Reagent (Roche). After selection with G418 (Wako), limiting dilutions were performed. A clone, which grew well in the orthotopic site was chosen and used as MUC1-transfected cell (SL4-MUC1). A clone transfected with empty plasmid vector (pDKOF), and showed similar *in vivo* growth to SL4-MUC1 was used as a control (SL4-mock).

### Orthotopic Tumor Transplantation

SL4-mock or SL4-MUC1 cells (1×10^6^ cells/50 µL) were injected into the space under the cecal serosa of B6 mice or MUC1.Tg mice. Two weeks after the injection, mice were sacrificed, and the weights of the ceca were measured.

### Flow Cytometric Analysis of Treg Cells

SL4-MUC1 cells were injected into the ceca of naïve B6 or MUC1.Tg mice. Five or ten days after injection, mice were sacrificed, and the cecal tumors were resected, minced, and washed with PBS. Spleens and MLNs were also collected from tumor bearing mice, and single cell suspensions were prepared. The fragments of tumor tissues were digested in RPMI-1640 containing 10% FCS, 200 U/ml collagenase (Wako), and 0.02% DNase I (Roche) and gently agitated at 37°C for 1 hour. The cells were passed through nylon mesh to eliminate the large debris and washed with PBS, and single cell suspensions were prepared. To examine IL-10 production, cells from the cecal tumors were stimulated with 20 ng/ml PMA (Sigma) and 1 µM ionomycin (Calbiochem) in the presence of 10 µg/ml brefeldin A (Sigma) for 4 hours. Cells were stained with PE-anti-CD4 (RM4-5, BioLegend) and biotinylated anti-CD25 (7D4, BD Bioscience) followed by APC-streptavidin (BioLegend) or PE-Cy7-streptavidin (BioLegend). After cell surface staining, cells were fixed and permeabilized using the Foxp3 staining kit (BioLegend) and stained with Alexa488-anti-Foxp3 (MF-14, BioLegend) and Alexa647-anti-Ki-67 (B56, BD Pharmingen) or APC-anti-IL-10 (JES5-16E3, BioLegend). Flow cytometric analysis was performed on a FACSAria (BD), and data were analyzed using FlowJo software (Tomy digital).

### The Winn Assay

For the induction of MUC1-specific T cells, B6 mice were immunized intradermally three times at weekly intervals in the forelimb with 25 µg of pCEP4 or pCEP4-MUC1 dissolved in 50 µL of Hanks’ Balanced Salt Solution. One week after the final MUC1 DNA vaccination, mice were sacrificed, and single cell suspensions were obtained from the spleens. After removal of erythrocytes, splenocytes were passed through a nylon wool column to enrich for T cells. These enriched T cells (3×10^6^ cells) were mixed with MUC1-transfected B16-F10 cells [35] (5×10^4^ cells) and injected subcutaneously into naïve B6 or MUC1.Tg mice. The survival rates of the mice were then determined (n = 5).

### Preparation of Treg Cells

Naïve B6 or MUC1.Tg mice were sacrificed, and single cell suspensions were obtained from the spleens, MLNs and peripheral lymph nodes. After removal of erythrocytes, cells were stained with PE-anti-CD4 (RM4-5) and biotinylated anti-CD25 (7D4) followed by APC-streptavidin. CD4^+^CD25^high^ cells were sorted using a FACSAria and used as Treg cells. CD4^+^CD25^−^ naïve T cells were also sorted and used as control cells.

### Suppression of T Cell Function by Treg Cells

VF5 cells, 13-mer MUC1-specific CD4^+^ T cell hybridoma cells, were a kind gift from Dr. Olivera J. Finn [32] and were used as MUC1-specific responder cells. For the preparation of OVA-specific CD4^+^ T cells, B6 mice were injected in the footpad and near the tail base with 100 µg of OVA protein (Sigma) mixed with CFA. Ten days after the injection, spleens and draining LNs were collected. CD4^+^ T cells from OVA-immunized mice or OT-II TCR transgenic mice were obtained by negative selection using a mixture of biotinylated mAbs against CD11b, Ly6G, DX5, B220, and CD8 (BioLegend) and streptavidin-microbeads (Miltenyi Biotec) on AutoMACS (Miltenyi Biotec) according to the manufacturer’s specifications. BM-DCs were generated as previously described [36]. Immature BM-DCs were pulsed with 20 µg/mL 14-mer MUC1 peptide (AHGVTSAPDTRPAP), 100 µg/mL OVA, or 0.5 µg/ml OVA peptide 323–339 (ISQAVHAAHAEINEAGR) and cultured for 18 hours. After washing with RPMI-1640, 2×10^4^ or 4.5×10^3^ BM-DCs were co-cultured with 1×10^5^ VF5 or OVA-primed CD4^+^ T cells or 4.5×10^4^ OT-II T cells in 96-well U-bottom plates. Differential numbers of Treg cells or naïve CD4^+^ T cells from B6 or MUC1.Tg mice were added to the culture system, and the suppressive capacity of each cell type was evaluated. Twenty-four hours for VF5 and OVA-primed CD4^+^ T cells or 48 hours for OT-II T cells after the co-culture, supernatants were collected, and IL-2 concentration was measured using the mouse IL-2 ELISA Ready-SET-Go! (eBioscience).

### Statistical Analysis

Statistical analysis for *in vivo* experiments shown in Fig. 1 and 2 was calculated on non-parametric Mann-Whitney U-test. Survival was calculated on a Kaplan-Meier survival plot and significance was assessed by the Logrank test. A student’s t test was used to determine statistical significance in the IL-2 ELISA assays shown in Fig. 5. Differences with *P*<0.05 were considered significant.
